# Determinants of Satisfaction With Hospital Online Appointment Service Among Older Adults During the COVID-19 Pandemic: A Cross-Sectional Study

**DOI:** 10.3389/fpubh.2022.853489

**Published:** 2022-02-18

**Authors:** Wenjia Li, Shengwei Shen, Jidong Yang, Jingyu Guo, Qinghe Tang

**Affiliations:** ^1^College of Communication and Art Design, University of Shanghai for Science and Technology, Shanghai, China; ^2^School of Medicine, Tongji University, Shanghai, China; ^3^School of Creativity and Art, Shanghai Tech University, Shanghai, China; ^4^Shanghai East Hospital, Tongji University, Shanghai, China

**Keywords:** pandemic, older adults, digital divide, Internet-based appointment service, satisfaction, aging design

## Abstract

**Background:**

How did older adults who had to use online medical service during the COVID-19 pandemic bridge the “digital divide”? Taking Internet-based appointment service (IBAS) as an example, this study aimed to investigate the subjective feelings of older adults and evaluate their user-satisfaction.

**Methods:**

This study was based on data from a questionnaire survey involving 325 outpatients 60 years old in shanghai during the COVID-19 pandemic. The satisfaction of IBAS was evaluated and compared from six domains including convenience, visiting time, correct identification of specialists, on-site assist service, COVID-19 prevention, and privacy protection. Logistic regression analysis was used to investigate the correlation between satisfaction and social factors.

**Results:**

No significant difference between older adults with or without previous experience using IBAS in terms of overall satisfaction. In the domain of operation difficulty (81.9 vs. 97.5%) and precise medicine (88.1 vs. 96.9%), such as correctly identifying the specialist, the satisfaction of previous user group was significantly higher than that of first-time user group. However, there was no significant difference in the remaining four domains between the two groups. Among the first time IBAS users, the satisfaction was higher than the walk-in registration they used before. Logistic regression revealed that some “intention to use IBAS”-associated social factors such as distance from the hospital, living status, and frequency of hospital visit, were related to the satisfaction of older adults.

**Conclusions:**

Driven by the external pandemic and internal intention, older adults would choose and manage network medical resources with their high satisfaction, which essentially demonstrates not only behavioral adjustment but also inner acceptance in older adults. Our findings support the need for promoting the driving force of older adults in using Internet-based medical service as well as transforming the design factors and behavior patterns.

## Introduction

COVID-19 has posed great challenges to public health around the globe since its onset from early 2020. Notably, various of epidemic prevention and control strategies have been extensively implemented long before the large-scale clinical application of vaccines and herd immunity could be realized in China (1). Targeted social distancing is the standard practice to influence infection spread, especially for vulnerable groups since COVID-19 is most likely to spread through close contact (2, 3). To eliminate the spread of the pandemic, effective social distancing was implemented in China since February 2020, which means not only keeping physical space between people but also avoiding unnecessary social contact (4, 5). Under such circumstances, diversified online services have been rapidly launched and developed in the form of “Internet-based” such as video conference, online shopping, and online medicine service, effectively avoiding crowd gathering and unnecessary contact (6–8).

**Figure 1:**
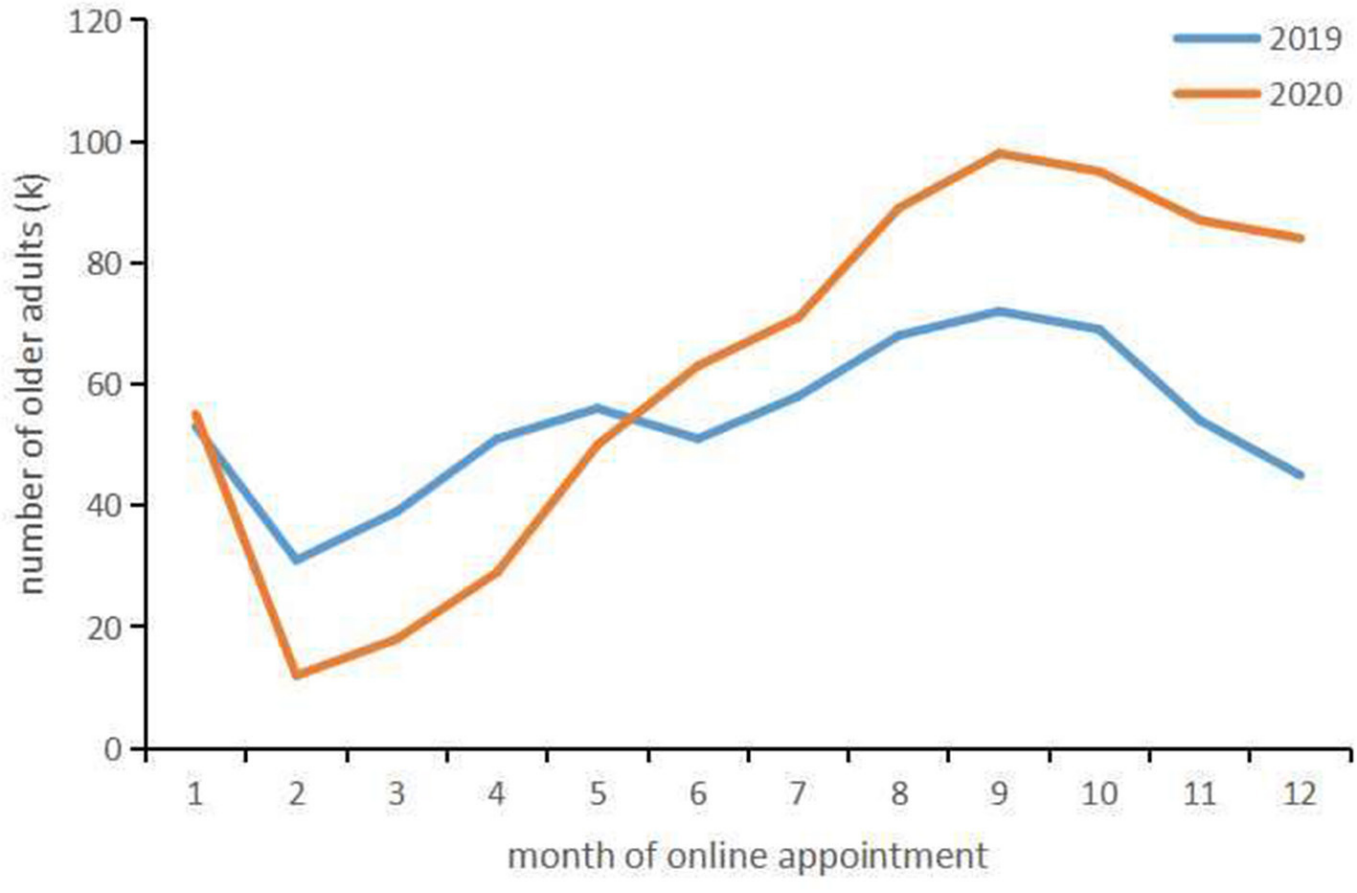
Trend in the number of older adults using online appointment in 2019 and 2020.

In the process of medical treatment, outpatient waiting time has been a well-known issue in China. The walk-in outpatients will experience triage, registration, payment and waiting before they can meet a physician where all the processes happen in outpatient's waiting area. The outpatient clinic becomes the place where patients are more likely to contract virus due to concentrated crowd and intensive contact. For this reason, more and more medical institutions implement appointment and registration process online in order to reduce the gathering of patients during the pandemic (9, 10). As an important part of online medical service, Internet-based appointment service (IBAS) provided by hospital, usually displaying the largest variety of information on a hospital website, such as disease categories and physician treatments, is informative (10, 11). The outpatients can complete all the appointment processes in advance through the network, arrange medical consultation or plan, and go to the clinic directly at the scheduled time without long stay in the waiting area. Although IBAS is not a compulsory measure, most outpatients choose it voluntarily during the pandemic. According to the data from one of the biggest online appointment and registration websites in China (www.yihu.com), from February to April in 2020, the number of online appointments declined compared with 2019 due to epidemic management and flow restriction in some of the hospitals. However, the number of online appointments surged after May 2020 compared with corresponding period of 2019. At the same time, the data also suggested that the number of older adults over 60 years old who used IBAS kept increasing in 2020, and it was significantly higher than that in the same period of 2019 (Figure 1).

Nevertheless, for older adults, the utilization of Internet-based medical and other social public services depends on the familiarity and mastery of the Internet and smart devices. The existence of “digital divide” in older adults is a universal global problem in the digital era (12, 13). Due to the weak ability of accepting and updating real-time information, which results in their disadvantages in learning skills of the Internet, older adults are often treated as the digital vulnerable group (14–16). In recent years, China has help older adults to lower the digital barrier and promote the Internet-based service. Even though knowledge of the IBAS was widespread, data shows that most elderly patients still choose walk-in registration before the pandemic, which makes IBAS difficult to implement and limited in its effect (17). As data mentioned above, some elderly began to use the IBAS for the first time due to epidemic control and personal protection. Different from the previous elderly IBAS users, these new users were with much stronger intention since they should make a choice to adapt to the life under the epidemic. How did they feel about their choice? Whether they could appreciate the convenience brought by Internet services? So far there have been few studies on the relationship between older adults and online medical service (18, 19). Most of them focus on the correlation between the network application and social attributes of older adults while less attention is paid to their internal demands and subjective feelings.

Our study intends to investigate the subjective feelings of these new Internet service elderly users and evaluate their satisfaction. And compared with older adults who had IBAS experience before the pandemic. The hypothesis of our study is that older adults who first used the IBAS during the pandemic are satisfied with the service, may have similar user experience compared with those who used IBAS before the pandemic. On the other hand, IBAS could benefit these new users in improving their familiarity with online medicine care service and digital health. The objectives are listed as follows: (a) Evaluate the understanding and feelings of older adults using IBAS during the pandemic; (b) Analyze social factors which might cause the difference in satisfaction; and (c) Explore older adults personal demands for online appointment. These will help to observe the degree of satisfaction in IBAS utilization from the intention perspective among the older population and explore an effective way for them to deal with the mental impact brought by the network application.

## Methods

### Questionnaire Design and Sample Selection

This is a cross-sectional study conducted in Shanghai East Hospital affiliated to Tongji University. The questionnaire was designed following literature research and expert interviews. A pilot survey was carried out before the formal survey. A total of 100 questionnaires were distributed to the qualified elderly respondents, and then 95 valid questionnaires were recollected. According to the pilot survey, some questions calling for further clarifications were modified, and the official version was issued. The questionnaire was divided into three parts. First, basic demographic information including gender, age, education background, medical insurance, assist mode of registration, and frequency of visit to hospital, the awareness of IBAS, and the reason for choosing IBAS. Second, satisfaction and scene investigation. A total of 16 questions were separated in six domains including: (a) convenience of IBAS system (interface settings, information input mode, online payment, and refund), (b) visiting time (time required for advance appointment, on-site waiting time, and total time spent), (c) precise medicine (scheduled time reservation, designated department or specialist, and required inspection item), (d) on-site assist service, (e) need for COVID-19 prevention, and (f) privacy protection and the multiple evaluation index of “overall satisfaction of this visit” was also included. The 16 qualitative indicators were all assessed by using the 5-point Likert scale to distinguish the evaluation of patients on the preference for different situations in medical experience. Likert scale is a tool to measure attitude, which has had a great impact on the field of social science since it was proposed. Likert scale ranges from “very dissatisfied” to “satisfied,” namely 1 for very dissatisfied, 2 dissatisfied, 3 basically satisfied, 4 for satisfied, and 5 for very satisfied (20). Third, satisfaction comparison. This part is only to be filled out by respondents of first using online appointment, which was intended to compare the use of previous walk-in appointment.

We started the study in January 2021 and instructed trained research assistants at IBAS waiting area to interview the current patients to complete the questionnaire. In China the “elder people” starts at 60 which is stipulated by the law on the protection of the rights and interests of older adults. The average life expectancy of China's population is over 70 years, but after the age of 60, the physique has declined significantly, and generally no longer undertakes physical work. Internationally, the starting age standard for the elderly in developed countries is 65, while that in developing countries is 60. China has adopted the standard of developing countries. In this study 60 years is used as the cut-off point for the sample. The inclusion criteria include: (1) Age ≥60 years old; (2) Ability to answer the questionnaire independently; (3) Informed consent (willing to participate in this study). The exclusion criteria include: (1) Patients with any medical disputes; (2) Severe emergency patients. The recruited patients were divided into two groups according to whether they first started using IBAS before or after February 1, 2020, when China began to initiate “social distancing” measures. The first-time user group included those who first used IBAS after this date, while the previous user group included those who had used IBAS before. According to Sun (21), the sample size needs to be 5–10 times the number of items in the survey, and allowing for 10–20% over-accrual at the same time to account for errors or potentially not evaluable feedbacks. Since our survey contains 16 items, the sample size should range from 88 to 192. Considering the limitation on budget and research staff of the study, a total of 374 elderly patients were recruited, and 348 of them completed the questionnaire. Excluding the questionnaires answered by caregivers, eventually 325 participants were selected. All questionnaires were administered anonymously to discourage an acquiescent or socially-desirable response bias.

### Ethical Requirements

The present study abided by the general ethical principles, respected patients' privacy, and obtained informed consent from every participant. Respondents were fully informed of the research purpose and content prior to study, and maintained the complete right of voluntary participation. Anonymity was used in the survey, and the information provided by the respondents was kept confidential and not used for other purposes except this study.

### Questionnaire Reliability and Validity Analyses

The reliability of the questionnaire was evaluated by most commonly used Cronbach's α to test its internal consistency. The validity of the questionnaire was evaluated through factor analysis, Kaiser Meyer Olkin (KMO) test and Bartlett's test were applied. The reliability and validity of six domains and 16 indicators evaluating the satisfaction of the research group and control group were respectively tested. The results showed that the Cronbach's α of intergroup variables was more than 0.9, indicating a good internal consistency. Moreover, KMO value of intergroup variables was also more than 0.9, and *P* value following Bartlett's test was significantly <0.001, further implying a good structural validity.

### Statistical Analyses

During data processing, satisfaction was converted into two categories: “very dissatisfied” and “dissatisfied” were defined as “dissatisfied” (assigned as 0) whereas “basically satisfied,” “satisfied,” and “very satisfied” were regarded as “satisfied” (assigned as 1). The overall satisfaction and the satisfaction of each domain were given a numerical value respectively. Chi-square test was used to compare the outcome of satisfaction between two groups, and logistic regression analysis was used to investigate how the satisfaction was associated with social factors including variables such as age, gender, distance from the hospital, living status, frequency of visits in 6 months, medical insurance, pension, and education background. *P* < 0.05 was considered to be statistically significant. SPSS 26.0 (IBM Corporation Armonk, NY, USA) was used to perform these analyses existing in this study.

## Results

### Descriptive Characteristics of Respondents

Among the 325 eligible respondents, the median age was 74 years (IQR: 72–79). In the first-time user group, 80 elderly persons (49.1%) live with their children or spouse while 83 elderly persons (50.9%) live alone. In contrast, 106 elderly persons (65.4%) live with their children or spouse while 56 elderly persons (34.6%) live alone in previous user group. Within 6 months, 49.1% and 56.8% respondents in first-time user group and previous user group visited medical institutions more than once. The learning approach in first-time user group were through children and community assistance (43.6%), on-site guidance (38%), and self-study (18.4%), while in the previous user group were self-study (61.1%), children and community assistance (25.3%), and on-site guidance (13.6%) (Table 1).

**Table 1:** The characteristics of respondents.

**Factors**	**First-time users** **(*****n*** **=** **163)**		**Previous users** **(*****n*** **=** **162)**	
	**Number**	**Proportion (%)**	**Number**	**Proportion (%)**
**Gender**				
Male	85	52.1	82	50.6%
Female	78	47.9%	80	49.4%
**Age**				
60–75	98	60.1%	93	57.4%
>75	65	39.9%	69	42.6%
**Distance to hospital**				
<30 min	79	48.5%	68	42.0%
>30 min	84	51.5%	94	58.0%
**Medical insurance**				
Yes	82	50.3%	83	51.2%
No	81	49.7%	79	48.8%
**Hospital visit within 6 months**				
0–1	83	50.9	70	43.2
>1	80	49.1	92	56.8
**Living status**				
Alone	83	50.9	56	34.6
With children	42	25.8	52	32.1
With spouse	38	23.3	54	33.3
**Pension**				
Yes	92	56.4	99	61.1
No	71	43.6	63	38.9
**Education**				
Below middle school	73	44.8	59	36.4
Middle school	42	25.8	52	32.1
College or above	48	29.4	51	31.5
**Learning approach**				
Self-study	30	18.4	99	61.1
Children or community	71	43.6	41	25.3
On-site guidance	62	38.0	22	13.6

### Satisfaction of Respondents With Online Appointment

There was no significant difference between the two groups in terms of overall satisfaction (*P* > 0.05). In the domain of operation difficulty (81.9 vs. 97.5%) and precise medicine (88.1 vs. 96.9%), such as correctly identifying the specialist, the satisfaction of previous user group was significantly higher than that of first-time user group. However, there was no significant difference in the remaining four domains between the two groups. Of note, the satisfaction with first IBAS user was only similar to the previous walk-in registration in the domain of on-site assist service (68.1 vs. 71.9%), but higher in the other five domains of using convenient degree, visiting time saving, epidemic prevention needs, precise medicine, and privacy protection (*P* < 0.05) (Table 2).

**Table 2:** The satisfaction of respondents with internet-based appointment service.

**Satisfaction domains**	**First-time users**			**Previous users**	***P* value**
	**Online service**	**Walk-in service**	***P* value**		
Convenience of operation system	131 (81.9)	107 (66.9)	0.02	156 (97.5)	<0.001
Visiting time	150 (93.6)	98 (61.3)	<0.001	148 (92.5)	0.658
Precise medicine	141 (88.1)	118 (73.8)	<0.001	155 (96.9)	0.0029
On-site assist service	110 (68.7)	101 (63.1)	0.228	115 (71.9)	0.54
COVID-19 prevention	155 (96.9)	78 (48.8)	<0.001	158 (98.8)	0.251
Privacy protection	142 (88.8)	131 (81.9)	<0.001	152 (95.0)	0.05
Total	130 (81.3)	89 (55.7)	<0.001	137 (85.6)	0.29

### Univariate Analyses of Risk Factors Influencing Satisfaction

The univariate analyses of the factors affecting satisfaction using IBAS were reported in Table 3. In the overall study population, those respondents over 75 years old, with more than 30 mins away from the hospital exhibited lower satisfaction (OR = 0.37, *P* < 0.05; OR = 0.51, *P* < 0.05). Meanwhile, the satisfaction of those college-educated elderly either living with children or visiting hospital more than once within 6 months was higher (OR = 5.05, *P* < 0.01; OR = 3.38, *P* < 0.01; OR = 2.51, *P* < 0.01). For those first-time users, distance to hospital, living status, and education were significantly related to dissatisfaction (*P* < 0.05). As for previous IBAS users, dissatisfaction was closely associated with the factors of age and education (*P* < 0.05).

**Table 3:** Univariate analyses of the factors influencing the satisfaction of older adults.

**Factors**		**Total (*****n*** **=** **325)**				**First-time users (*****n*** **=** **163)**				**Previous users (*****n*** **=** **162)**			
		***n***	**Dissatisfied (%)**	**OR (95% CI)**	***P* value**	***n***	**Dissatisfied (%)**	**OR (95% CI)**	***P* value**	***n***	**Dissatisfied (%)**	**OR (95% CI)**	***P* value**
Age	60–75	191	12.0			98	17.3			93	6.5		
	>75	134	23.1	0.37 (0.19, 71.4)	**0.03**	65	21.5	0.53 (0.21, 1.35)	0.191	69	24.6	0.21 (0.07, 5.67)	**0.003**
Gender	Male	167	16.8			85	20			82	13.4		
	Female	158	15.6	0.78 (0.40, 1.50)	0.452	78	16.7	0.81 (0.31, 2.08)	0.661	80	15	0.70 (0.25, 1.96)	0.502
Distance to hospital	<30 min	147	16.3			79	13.9			68	19.1		
	>30 min	178	16.9	0.51 (0.25, 0.98)	**0.045**	84	23.8	0.36 (0.13, 0.90)	**0.034**	94	10.6	0.63 (0.22, 1.72)	0.378
Medical insurance	Yes	165	17			82	19.5			83	14.5		
	No	160	16.3	1.05 (0.54, 2.04)	0.891	81	18.5	0.76 (0.30, 1.93)	0.569	79	13.9	1.55 (0.53, 4.60)	0.421
Hospital visit within six months	0–1	153	20.9			83	24.1			70	18.5		
	>1	172	11.6	2.51 (1.30, 4.98)	**0.007**	80	12.5	2.21 (0.83, 5.00)	0.093	92	10.8	2.44 (0.89, 7.00)	0.086
Living status	Alone	139	25.9			83	36.1			56	10.7		
	With children	94	8.6	3.38 (1.45, 8.72)	**0.007**	38	5.3	6.50 (1.80, 32.59)	**0.009**	52	11.5	2.06 (0.56, 8.44)	0.288
	With spouse	92	18.4	1.96 (0.89, 4.51)	0.102	42	11.9	2.32 (0.78, 8.03)	0.150	54	22.2	1.62 (0.47, 5.89)	0.450
Pension	Yes	191	17.3			92	19.6			99	15.1		
	No	134	16.4	0.91 (0.47, 1.74)	0.771	71	18.3	1.55 (0.53, 4.60)	0.781	63	14.2	0.88 (0.33, 2.37)	0.805
Education	Below middle school	132	22.7			73	24.6			59	20.3		
	Middle school	94	20.2	1.00 (0.49, 2.05)	0.997	42	23.8	4.98 (1.41, 23.97)	0.514	52	17.3	1.50 (0.53, 4.43)	0.448
	College or above	99	5.1	5.05 (1.93, 15.92)	**0.002**	48	6.3	0.72 (0.26, 1.98)	**0.023**	51	3.9	8.13 (1.75, 60.61)	**0.016**

*All the significant factors (P <0.05) are in bold*.

## Discussion

As people gradually get used to life in the pandemic, whether the “digital right” of older adults with digital barriers that exists widely in these people can be guaranteed, and whether they can get high-quality Internet-based public services especially medical and health care, are the issues worthy of attention from the administration and medical institutions (13, 22, 23). This study explored the evaluation of older adults who started using IBAS during the COVID-19 pandemic. The results showed that their satisfaction was commonly higher than walk-in registration they chose before (*P* < 0.05), and there was no significant difference with those elderly with that of previous IBAS users (81.3 vs. 85.6%, *P* > 0.05). Our study shows that on the premise of the continuous lowering of the technical threshold, the definition of digital barriers is not only limited to the technical level, but also including the low usage intention of Internet. While the pandemic becomes the norm of life, the change of life style has forced those potential Internet users among older population to break their thinking inertia and make some breakthroughs. With increased intension, they will manage to solve potential technical difficulties and realize the convenience brought by the Internet services.

The IBAS has potential benefits, which can greatly reduce the burden of time and energy paid by older adults and their caregivers. However, the utilization of IBAS in older adults is generally low before the COVID-19 pandemic in China. The main using obstacles are summarized as follows: insufficient awareness; incompetence in using the system; limited choice of treatment time, doctors, and departments; inability to complete special services *via* the Internet; difficulty in online payment; and individual willingness. In an effort to solve these problems, the administration, medical institutions, communities as well as mass media, have provided convenient measures for older adults, such as setting up access to IBAS through the public network platform, continuously optimizing and simplifying the operation procedure, and specifically adjusting the display of font, text, and audio-visual input for older persons (24). In addition, the communities and medical institutions have provided a lot of training opportunities to help them understand and master how to pay online. The implementation of above measures has greatly lowered the threshold for older adults to access Internet-based medical services. Nevertheless, the traditional way of queue registration or walk-in service is still the first choice for quite a lot of elderly patients who are unfamiliar or unwilling with the Internet-based service. In this study, 71.7% of the first-time users acknowledged that even if they knew about IBAS and online payment previously, they still did not choose them for “personal reasons,” including usage and payment habits. Meanwhile, up to 92.0% of all these patients identified the concern about increased infection potentials caused by prolonged staying in the hospital as the primary reason for choosing IBAS. Taken together, these facts suggest that the needs for epidemic prevention and control and personal protection can be considered as the external reasons for older adults to use online medical services, but in older adults with potential ability to use Internet-based service, “low usage intention” becomes an important factor for them to make choices.

The phenomena of “lack of intention” can be explained by the “lack of confidence” in Internet use among older adults. The application of digital technology facilitates public service, but there is still explicit or implicit social exclusion for older adults who fail to utilize digital technology effectively (25). Internet media and its content producers have long since paid more attention to meet the needs of the mainstream consumers while ignored the needs of older adults (14). Although various institutions have tried their best to solve this tough problem, there is still a serious lack of designs that suit the needs of the aging population, which objectively increases the difficulty for older adults to accept new information and recognize new technology. These obstacles further diminish their confidence to try new technology, making older adults harder to integrate into the informational society. Meanwhile, due to the decline of physical and cognitive ability along with aging, older adults tend to panic more easily in the face of technological changes, and may choose to keep their original living habits. The designer of IBAS should fully consider the use needs of different groups of people at different levels, solve the problems of users when operating the software effectively, develop and design the interface for the convenience of older adults according to their physical and psychological characteristics, and fully pay attention to the interface structure, interface layout element location and visual process. By optimizing the design, older adults would have a voice in public terminals and appreciate the usability and accessibility of IBAS. Meanwhile, a “fault-tolerant” interaction design mechanism should be established for older adults, which can be revoked in time after their misoperation. It would effectively alleviate the anxiety and fear of older adults for IBAS participation.

Using Internet to solve problems has become a social development trend and one of the major life skills (26). Internet use had a certain positive impact on the physical and psychological healing of older adults, and had a certain effect on their self-cognition, social interaction, life satisfaction and self-confidence (27, 28). As we can see that, different from those previous IBAS users, the new IBAS users may not had advantages in self-learning skills. Most of the them (81.6%) seek to solve the technical problems through community, children or on-site assistance. Our study confirmed that once older adults are willing to use the network, they will try to get the technical support through the diversified approaches. Previous study about the Internet support for older adults mainly focuses on “digital feedback” from their children and other family members (29). Nowadays older adults are trying more approaches to master network skills, such as peer support. For older adults peer groups, the similarities in experience and learning speed make them an easier counterpart to communicate with and obtain empathy from compared with their children. They can provide emotional and informational support more easily with geographical and psychological proximity. Therefore, friends, colleagues, neighbors, and other peer groups, as the important sources of social support for older adults, often become an alternative to intergenerational support.

By analyzing the factors that influencing the satisfaction of IBAS, we found that satisfaction-associated social factors were also related to the “intention to use” of older adults (30). In our study, patients who live further away from the hospital are more willing to use IBAS to achieve convenient medical care, so as to shorten the time spent. Compared with older adults who live with their children or spouse, older adults who live alone had lower satisfaction with IBAS, suggesting that older adults for whom can get appropriate digital assistance are more willing to choose online health care and have higher satisfaction. Compared with older adults who visited the hospital 0–1 within 6 months, those who visited the hospital more than once preferred IBAS. The re-visiting patients are familiar with the triage and visiting doctors, which helps to make appointments much smoother, and subjectively promotes the using intention. In summary, these above results show that it is possible to improve the usage intention to Internet-based medical services more effectively through analyzing the big data from specific groups of older adults, taking targeted measures to formulate medical services and assistance strategies, and providing personalized services.

The limitations of this study are listed as follows. First, the survey was conducted in a large tertiary public hospital located in Shanghai. Shanghai is highly urbanized with high average education level, low COVID-19 infection, and strong awareness and voluntary compliance with the provisions of epidemic prevention among its residents. Hence, the generalizability of the results from this study in other areas of the country is questionable. Second, the study focused on the population who chose IBAS, but did not analyze the population who still chose traditional methods of appointment. Therefore, all the conclusions of our study should be further investigated in prospective research with more diversified groups in the coming years.

## Conclusions

Driven by the external pandemic, older adults tend to choose and manage online medical resources and their network skill has been improved, which essentially demonstrates not only behavioral adjustment but also intrinsic intention ascension. Therefore, it will be an effective strategy to improve the long-term development of Internet based medicine through promoting the driving force of older adults as well as transforming their behavior patterns and habit formation. Today, with the increasing aging population, the problem of digital divide among older adults needs to be solved urgently. The administration and society should adhere to the principle of age-appropriate in top-level design and product design, construct a digital divide bridging mechanism, and create a digital inclusive daily life. By enjoying the convenience and efficiency of the digital and Internet based service, the older population would achieve the “active aging” more smoothly. Online medical resources should fully protect the rights of older adults groups to enjoy social public services, and the “age-friendly” digital strategy should be included in the development plan of medical informatization.

## Data Availability Statement

The raw data supporting the conclusions of this article will be made available by the authors, without undue reservation.

## Ethics Statement

This study was performed in line with the principles of the Declaration of Helsinki, revised version from 2000. The Ethics Committee of Shanghai East Hospital approved this study (Ref: EHE 2020-27).

## Author Contributions

WL and JY designed the study question, performed the statistical analyses, and wrote the original version of the manuscript. WL, JG, and SS performed the investigation. WL and QT designed the questionnaire and tables. QT was responsible for the overall supervision of the study and the revision of the manuscript. All authors read and approved the final manuscript.

## Funding

This study was sponsored by Shanghai Pujiang Program (Grant No. 21PJC087).

## Conflict of Interest

The authors declare that the research was conducted in the absence of any commercial or financial relationships that could be construed as a potential conflict of interest.

## Publisher's Note

All claims expressed in this article are solely those of the authors and do not necessarily represent those of their affiliated organizations, or those of the publisher, the editors and the reviewers. Any product that may be evaluated in this article, or claim that may be made by its manufacturer, is not guaranteed or endorsed by the publisher.
